# COVID-19 cluster identification and support vector machine classifier model construction using global healthcare and socio-economic features

**DOI:** 10.1017/S0950268823001383

**Published:** 2023-08-30

**Authors:** Soumya Kanti Guha, Sandip Sadhukhan, Sougata Niyogi

**Affiliations:** 1 Department of Computer Application, Dinabandhu Andrews Institute of Technology and Management, Kolkata, India; 2 Department of Medical Lab Technology, Dinabandhu Andrews Institute of Technology and Management, Kolkata, India

**Keywords:** clinical management, coronaviruses, healthcare outcomes, mortality rates, predictive modelling, support vector machine

## Abstract

Coronaviruses of the human variety have been the culprit of global epidemics of varying levels of lethality, including COVID-19, which has impacted more than 200 countries and resulted in 5.7 million fatalities as of May 2022. Effective clinical management necessitates the allocation of sufficient resources and the employment of appropriately skilled personnel. The elderly population and individuals with diabetes are at increased risk of more severe manifestations of COVID-19. Countries with a higher gross domestic product (GDP) typically exhibit superior health outcomes and reduced mortality rates. Here, we suggest a predictive model for the density of medical doctors and nursing personnel for 134 countries using a support vector machine (SVM). The model was trained in 107 countries and tested in 27, with promising results shown by the kappa statistics and ROC analysis. The SVM model used for predictions showed promising results with a high level of agreement between actual and predicted cluster values.

## Introduction

Three major outbreaks caused by coronaviruses have affected the world: SARS in 2002, MERS in 2012, and COVID-19 in 2019. While mortality rates associated with pandemics can fluctuate depending on individual factors, such as age and overall health status, the lethality of these viruses can also shift as medical knowledge and treatment methods advance ([1–4], https://www.worldometers.info/coronavirus/). Considering the situation, the World Health Organization (WHO) affirmed COVID-19 as a global public health emergency [5, 6] in March 2020. The World Health Organization (WHO) and the Centers for Disease Control and Prevention (CDC) have been actively providing guidance and recommendations to prevent the spread of these viruses and minimise the risk of complications. Through adherence to the guidelines set forth by these organisations, individuals can play a crucial role in mitigating the transmission of these viruses, thereby safeguarding themselves and others from the deleterious effects of the pathogens [7]. Pneumonia is a prevalent cause of hospitalisation, characterised by symptoms such as fever, cough, dyspnoea, and chest pain. In severe instances, this condition can culminate in mortality among affected patients [8]. Rendering high-quality medical care to critically ill patients necessitates a robust and functioning medical infrastructure. Clinical management of COVID-19 patients mandates access to hospital beds, well-equipped mechanical ventilation devices, N95 respirators, isolation gowns, gloves, and trained medical professionals [9–11]. Higher COVID-19 federated death has been found to be correlated with depleted hospital resources [12, 13]. COVID-19 has shown differential effects depending on disparate age groups [14, 15]. Elderly people (>70 yrs) are more susceptible to different health complications [16]. Diabetes is recognised as a substantial risk factor for severe illness and mortality among hospitalised COVID-19 patients [3, 17–20]. The 1918 influenza pandemic resulted in a reduction of up to 11.8 years in life expectancy at birth in the United States. Similarly, the Ebola virus outbreak of 2014 had a substantial effect, reducing life expectancy by 1.6 to 5.6 years in Liberia. These clearly underscore the profound and catastrophic impact that pandemics can have on communities. By implementing measures to enhance accessibility to vaccines and treatments, it is feasible to diminish the impact of future pandemics and safeguard public health [21, 22]. The increased mortality rate associated with COVID-19 has the potential to result in a significant loss of life expectancy. COVID-19 has already claimed the lives of millions of people worldwide, and its impact on life expectancy will depend on a number of factors, including the number of deaths, the age distribution of deaths, and trends in mortality from other causes [23]. The Preston curve shows that, on average, people in wealthier countries have higher life expectancies and lower rates of adverse health outcomes and mortality. This relationship is largely because wealthier countries tend to have better-developed health systems and more resources to invest in public health, medical research, and disease prevention and treatment [24, 25]. Gross domestic product (GDP) is a measure of a country’s economic output and is used to estimate the aggregate amount spent on final goods and services. In theory, higher GDP should result in better living standards, including better education and public health programmes, which can lead to improved life expectancy. However, the relationship between GDP and life expectancy is complex and can be influenced by many factors, such as income distribution, access to quality health care, and the prevalence of disease [25–30]. Early on in the COVID-19 pandemic, clinical classifications of the disease were primarily based on clinical signs and symptoms, as well as radiological findings from chest imaging. However, as our understanding of COVID-19 has evolved the classification of the disease and disease-related outcomes has become more nuanced and now incorporates additional factors, such as the presence of comorbidities, disease severity, and the stage of the disease. By continuing to collect and analyse data on the disease, we can further improve our understanding of COVID-19 and enhance our ability to respond to future outbreaks [31, 32].

Our study found that we can logically create four clusters of 134 countries based on six parameters: death rate, age older than 70, GDP per capita, diabetes prevalence, hospital beds per thousand, and life expectancy. The initial six-parameter data were scaled and analysed (dimensionality reduced) through PCA and later applied to K-means. Further programmatically we developed an SVM model to train and test it on varied six-parameter country data. The model predicted cluster values, and the actual cluster values were compared using kappa statistics. The ROC curve showed the significant effectiveness of the SVM model.

## Methods

### Study design and data collection

The data collected from ‘Our World in Data’ and ‘World Health Statistics 2020’ provide valuable insights into the healthcare workforce and resources across different countries. This information is useful for decision-makers and healthcare organisations in identifying areas for improvement and allocating resources where they are needed the most. Our study collected data from 211 countries, focusing on six parameters, namely death rate, age older than 70 years, GDP per capita, diabetes prevalence, hospital beds per thousand, and life expectancy. However, it is important to note that the data have a cut-off date of 12 May 2022.

### Screening

The selection of data is a crucial step in any study as it impacts the reliability and final outcome of the results. For our study, we collected data from 211 countries, but only 158 had complete data on the selected six parameters. To reduce the influence of population size, we applied a population filter (Pf) to consider only countries with a population of over 1 million, resulting in 134 countries for our final analysis. Having a clear set of criteria for selecting data is important, although it does not guarantee the quality or accuracy of the research. Using a population filter, we aimed to minimise the risk of bias and increase the generalisability of our results, which can be confidently used to make informed decisions and recommendations.

### K-means clustering

Clustering algorithms can help identify hidden features in random data sets, providing a structural identity to the data by grouping similar data points together. K-means is a popular unsupervised learning algorithm that can be easily implemented to cluster data points. In our study, we used K-means to analyse data from 134 countries based on six parameters. We applied three methods – elbow, silhouette, and gap statistic – to determine the optimal number of clusters (K). We found that K = 4 using these methods and then normalised the data and performed principal component analysis (PCA) before applying K-means. This allowed us to assign each of the 134 countries a cluster number from 1 to 4. We used the NumPy, Pandas, Matplotlib, and scikit-learn Python packages to run the iterative algorithm for the elbow, silhouette, and gap statistic methods. While clustering does not guarantee the accuracy or quality of research, it provides a way to identify and group similarities in large data sets in an unsupervised manner.

### Supervised classification model

Predictive modelling using classification is a wonderful mathematical process used to predict future events or outcomes by analysing patterns in a given data set. These classification methods programmatically give rise to various supervised machine-learning algorithms. The support vector machine (SVM) technique is one such example, which ensures more effectiveness in high-dimensional spaces yet being stable. Hence, we chose the non-linear SVM technique as it precisely uses hyper-planes (lines or decision boundaries) with various kernel functions to classify data that lack linear separation, hence offering better prediction and less error.

First, we use the scaling technique on the six-parameter data and apply the principal component analysis (PCA) technique to reduce the data dimension. Once we receive the well-defined first two principal components of PC1 and PC2, we divide the 134 data sets of PC1 and PC2 into test and training data sets using the Pareto principle of the 80:20 rule [33]. Hence, we had 107 countries grouped under the training set and 27 countries featured under the testing set. Next, we developed a Python program to implement a predictive model using the SVM algorithm with the Gaussian kernel radial basis function. The model was trained using the training set (107 countries) to identify the correct clusters that a country originally belonged to. From K-means, we already had the targets identified as cluster numbers 1 to 4 assigned to every country based on the previously discussed six-parameter scale. Once training was finished, we programmatically applied the testing set to test the model. The model predicted a cluster each out of 1 to 4 for all the 27 countries. Next, we compared this predicted cluster number obtained from our SVM-trained model with the actual cluster number identified by the K-means clustering technique earlier. We applied kappa statistics to establish a substantial level of agreement between the actual and predicted cluster values for all the test data. The receiver operating characteristic (ROC) curve plotted for all four clusters showed the promising outcome and usefulness of this SVM model in general.

### Statistics

Welch’s t-test is an appropriate test to use when the sample sizes are unequal, and the variances between the two groups are also unequal. Most of our clusters have unequal sample sizes. Using a 5% significance level and marking statistical significance with a *P*-value less than 0.001, type I error can be minimised. In our study, statistical significance was marked by ‘***’ with a ‘*P*’ value <0.001.

## Results

### K-means cluster analysis of 134 countries

**Figure 1.**
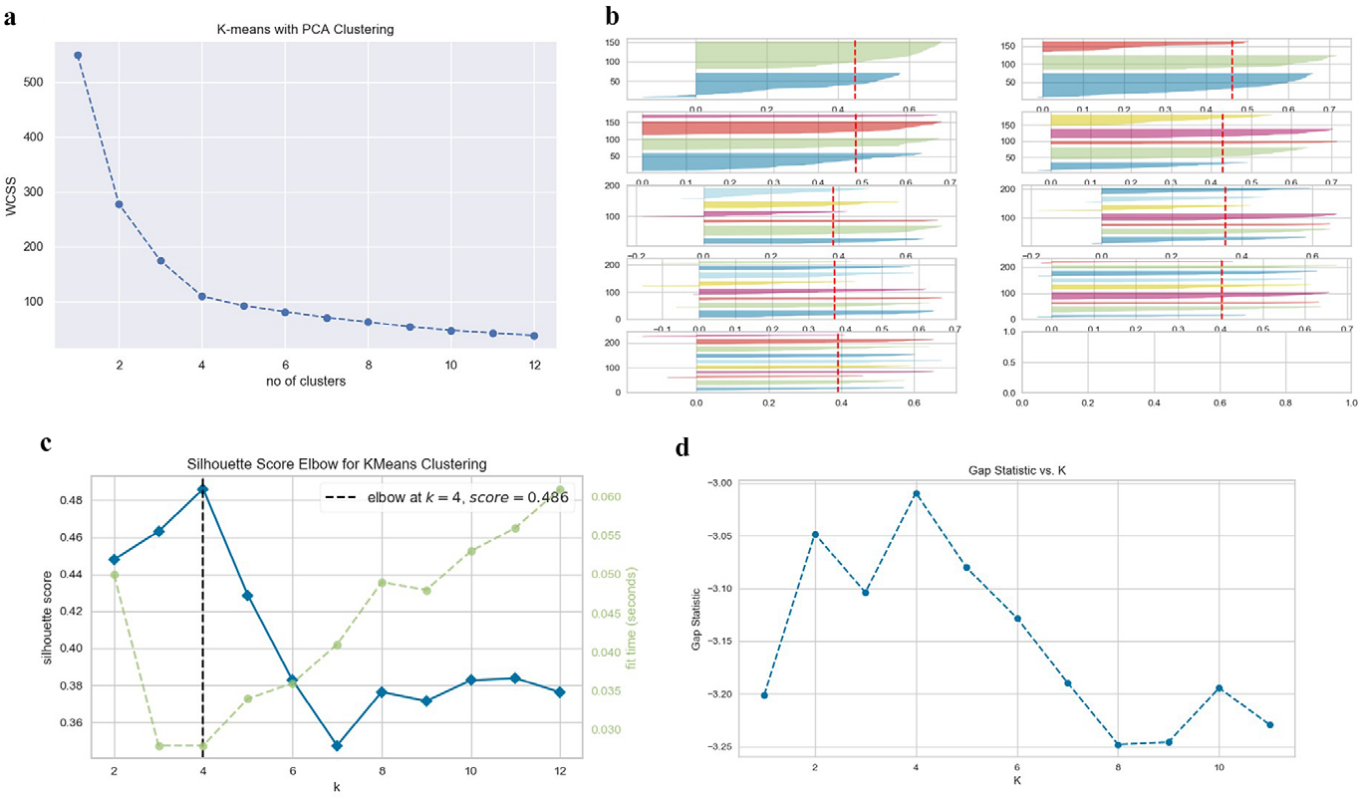
Selection of a number of clusters. Elbow (a), silhouette (b) & (c), and gap statistic (d) to find the probable number of clusters (K).

**Figure 2.**
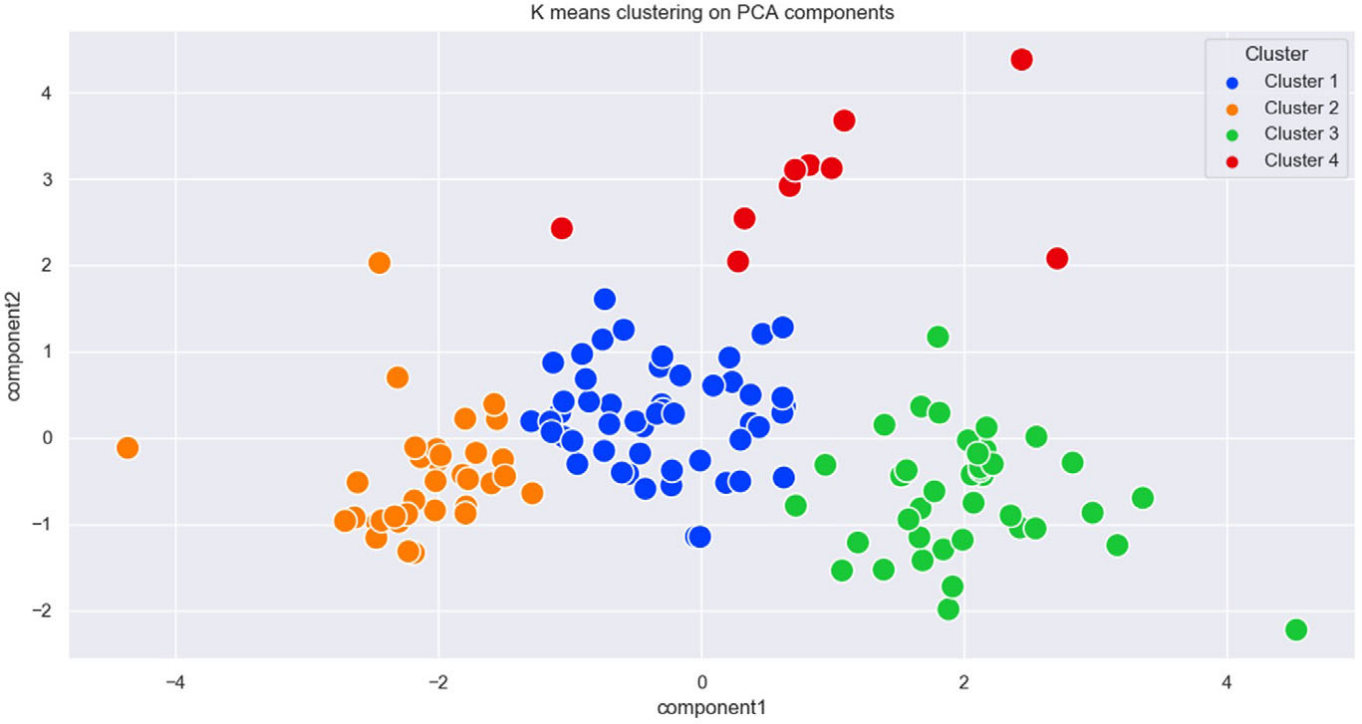
K-means clustering data. K-means clustering on PCA components was carried out in 134 countries using different parameters.

COVID-19, since its inception, has created a huge impact on global health. Total case fatality has escalated from one million in November 2020 to more than 6 million by April 2022. We selected 134 countries by applying the six-parameter scale mentioned earlier and performed PCA and K-means clustering. Initially, elbow, silhouette, and gap statistics were performed to determine optimal cluster numbers and consistently it was found to be 4 (Figure 1a–d). Next, we applied PCA first and then K-means clustering to classify these countries. Four distinct clusters were obtained, namely clusters 1, 2, 3, and 4 (Figure 2). In these four clusters (clusters 1, 2, 3, and 4), we had 51, 34, 38, and 11 countries listed, respectively (Table 1).

**Table 1.** List of clustered countries

Cluster 1	Cluster 2	Cluster 3	Cluster 4
Tajikistan	Eritrea	Estonia	Mauritius
Timor	Central African Republic	Latvia	Qatar
Nicaragua	Djibouti	Finland	Bahrain
Gabon	Equatorial Guinea	New Zealand	Oman
Uzbekistan	Liberia	Uruguay	Kuwait
Jamaica	Niger	Belarus	United Arab Emirates
Kyrgyzstan	Benin	Slovenia	Saudi Arabia
Trinidad and Tobago	Gambia	Norway	Singapore
El Salvador	Burundi	Lithuania	Egypt
Botswana	Burkina Faso	Ireland	Malaysia
Albania	Togo	Croatia	
Algeria	Guinea	Denmark	
Dominican Republic	Mali	Sweden	
Venezuela	Haiti	Slovakia	
Libya	Tanzania	Bulgaria	
Mongolia	Madagascar	Switzerland	
North Macedonia	Eswatini	Israel	
Armenia	Yemen	Hungary	
China	Laos	Austria	
Honduras	Cameroon	Greece	
Moldova	Ghana	Belgium	
Azerbaijan	Malawi	Portugal	
Costa Rica	Cambodia	Czechia	
Panama	Mozambique	Canada	
Bosnia and Herzegovina	Uganda	Romania	
Nepal	Sudan	Australia	
Lebanon	Zambia	Netherlands	
Sri Lanka	Zimbabwe	Japan	
Paraguay	Kenya	Ukraine	
Guatemala	Afghanistan	Poland	
Morocco	Ethiopia	Argentina	
Bolivia	Myanmar	South Korea	
Jordan	Pakistan	Spain	
Kazakhstan	South Africa	Italy	
Georgia		United Kingdom	
Tunisia		Germany	
Ecuador		France	
Bangladesh		Russia	
Iraq		United States	
Thailand			
Philippines			
Chile			
Vietnam			
Colombia			
Iran			
Indonesia			
Peru			
Turkey			
Mexico			
India			
Brazil			

### Cluster-wise distribution of divergent parameters

**Figure 3.**
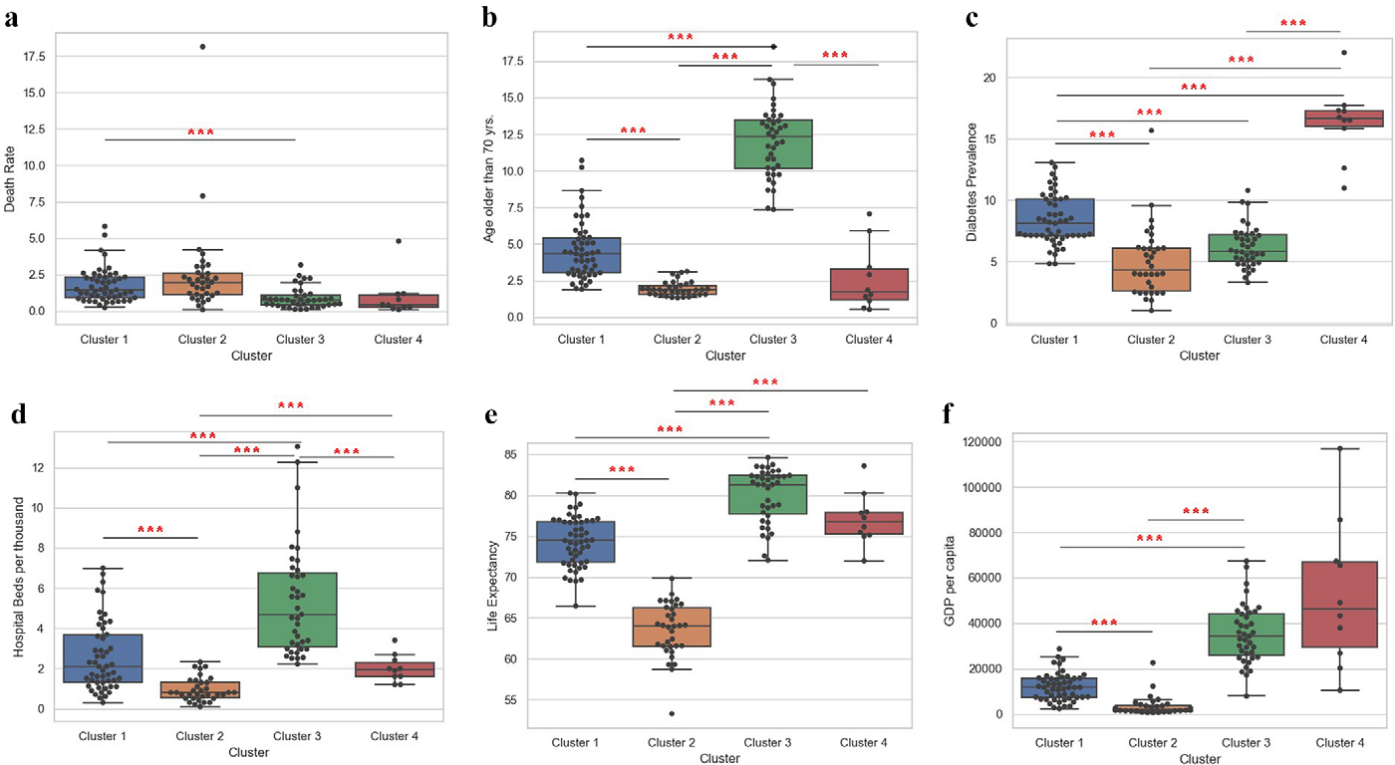
Cluster-wise distribution of different parameters. Death rate (a), age older than 70 yrs. (b), diabetes prevalence (c), hospital beds per thousand (d), life expectancy, (e) and GDP per capita (f) were plotted for all the clusters.

In our study, we have considered a six-parameter scale, namely death rate, age older than 70 years, GDP per capita, diabetes prevalence, hospital beds per thousand, and life expectancy across 134 selected countries. The death rate emphasises the ratio of deaths to the population of a particular area or during a particular period of time. All 134 countries have divergent deaths in our study. The parameter of age older than 70 years gives us a good knowledge of country-wise older adults who have the risk of getting COVID-19 due to their frail health. The parameter ‘age older than 70 years’ has comparatively high values for cluster 3. The trend decreases with clusters 1, 2, and 4, respectively. The diabetes prevalence parameter takes care of the strong probability of an individual experiencing comorbidity during the COVID-19 infection. Diabetes prevalence predominantly has high values for cluster 4, more than the maximum values of clusters 1, 2, and 3. The number of hospital beds per thousand people is an important metric that provides insight into a country’s healthcare system and its ability to provide adequate inpatient care. During the COVID-19 pandemic, the number of hospital beds per thousand people became even more important, as countries around the world struggled to provide enough hospital beds to care for the increasing number of patients with COVID-19. The parameter of hospital beds per thousand shows maximum variance for cluster 3, followed by clusters 1, 4, and 2. The median values of clusters 1 and 4 are similar. Cluster 2 suggests high overall data density. The parameter of life expectancy of any country establishes a statistical measure of the average time a citizen is expected to live, based on other demographic factors. Life expectancy for all four clusters is quite high though different. Data vary from 58 to 85 for all 134 countries. The median of cluster 3 is more than 80, followed by clusters 4 and 1, nearing 75 (Figure 3). Cluster 2 has a median value of less than 65. GDP per capita gives us a comprehensive impression of a country’s medical infrastructure, existing expenses, and future vision of health care. GDP per capita is most varied in cluster 4 followed by cluster 3. The trend decreases with clusters 1 and 2, respectively. Cluster 1 suggests high overall data density. All cluster-wise p-values for different parameters are given in Table 2.

**Table 2.** List of statistical parameters for different conditions

C1 vs C2 (death rate)	Value	C1 vs C2 (hospital bed)	Value	C1 vs C2 (life expectancy)	Value	C1 vs C2 (GDP per capita)	Value	C1 vs C2 (age > 70 yrs.)	Value	C1 vs C2 (diabetes prevalence)	Value
df	39	df	65	df	67	df	83	df	59	df	55
P(T < =t) one-tail	8.42X10^−02^	P(T < =t) one-tail	2.37 X10^−08^	P(T < =t) one-tail	1.42 X10^−23^	P(T < =t) one-tail	1.03 X10^−11^	P(T < =t) one-tail	4.47 X10^−13^	P(T < =t) one-tail	6.98 X10^−08^
P(T < =t) two-tail	1.68 X10^−01^	P(T < =t) two-tail	4.74 X10^−08^	P(T < =t) two-tail	2.84 X10^−23^	P(T < =t) two-tail	2.05 X10^−11^	P(T < =t) two-tail	8.93 X10^−13^	P(T < =t) two-tail	1.40 X10^−07^

C1 vs C3 (death rate)	Value	C1 vs C3 (hospital bed)	Value	C1 vs C3 (life expectancy)	Value	C1 vs C3 (GDP per capita)	Value	C1 vs C3 (age > 70 yrs.)	Value	C1 vs C3 (diabetes prevalence)	Value
df	85	df	61	df	78	df	51	df	73	df	87
P(T < =t) one-tail	1.86X10^−05^	P(T < =t) one-tail	3.15X10^−07^	P(T < =t) one-tail	1.01X10^−12^	P(T < =t) one-tail	1.95X10^−14^	P(T < =t) one-tail	5.60X10^−25^	P(T < =t) one-tail	1.46X10^−07^
P(T < =t) two-tail	3.72X10^−05^	P(T < =t) two-tail	6.29X10^−07^	P(T < =t) two-tail	2.01X10^−12^	P(T < =t) two-tail	3.90X10^−14^	P(T < =t) two-tail	1.12X10^−24^	P(T < =t) two-tail	2.91X10^−07^

C1 vs C4 (death rate)	Value	C1 vs C4 (hospital bed)	Value	C1 vs C4 (life expectancy)	Value	C1 vs C4 (GDP per capita)	Value	C1 vs C4 (age > 70 yrs.)	Value	C1 vs C4 (diabetes prevalence)	Value
df	12	df	36	df	12	df	9	df	12	df	11
P(T < =t) one-tail	5.79X10^−02^	P(T < =t) one-tail	4.37X10^−02^	P(T < =t) one-tail	1.40X10^−02^	P(T < =t) one-tail	1.72X10^−03^	P(T < =t) one-tail	1.18X10^−02^	P(T < =t) one-tail	2.68X10^−06^
P(T < =t) two-tail	1.16X10^−01^	P(T < =t) two-tail	8.74X10^−02^	P(T < =t) two-tail	2.80X10^−02^	P(T < =t) two-tail	3.45X10^−03^	P(T < =t) two-tail	2.35X10^−02^	P(T < =t) two-tail	5.36X10^−06^

C2 vs C3 (death rate)	Value	C2 vs C3 (hospital bed)	Value	C2 vs C3 (life expectancy)	Value	C2 vs C3 (GDP per capita)	Value	C2 vs C3 (age > 70 yrs.)	Value	C2 vs C3 (diabetes prevalence)	Value
df	36	df	42	df	70	df	47	df	42	df	52
P(T < =t) one-tail	2.27X10^−03^	P(T < =t) one-tail	8.76X10^−13^	P(T < =t) one-tail	2.16X10^−32^	P(T < =t) one-tail	3.08X10^−19^	P(T < =t) one-tail	2.28X10^−27^	P(T < =t) one-tail	1.64X10^−02^
P(T < =t) two-tail	4.55X10^−03^	P(T < =t) two-tail	1.75X10^−12^	P(T < =t) two-tail	4.32X10^−32^	P(T < =t) two-tail	6.16X10^−19^	P(T < =t) two-tail	4.56X10^−27^	P(T < =t) two-tail	3.27X10^−02^

C2 vs C4 (death rate)	Value	C2 vs C4 (hospital bed)	Value	C2 vs C4 (life expectancy)	Value	C2 vs C4 (GDP per capita)	Value	C2 vs C4 (age > 70 yrs.)	Value	C2 vs C4 (diabetes prevalence)	Value
df	34	df	13	df	15	df	9	df	9	df	14
P(T < =t) one-tail	1.44X10^−02^	P(T < =t) one-tail	3.43X10^−04^	P(T < =t) one-tail	3.17X10^−09^	P(T < =t) one-tail	5.05X10^−04^	P(T < =t) one-tail	1.76X10^−01^	P(T < =t) one-tail	1.78X10^−08^
P(T < =t) two-tail	2.88X10^−02^	P(T < =t) two-tail	6.86X10^−04^	P(T < =t) two-tail	6.34X10^−09^	P(T < =t) two-tail	1.01X10^−03^	P(T < =t) two-tail	3.51X10^−01^	P(T < =t) two-tail	3.55X10^−08^

C3 vs C4 (death rate)	Value	C3 vs C4 (hospital bed)	Value	C3 vs C4 (life expectancy)	Value	C3 vs C4 (GDP per capita)	Value	C3 vs C4 (age > 70 yrs.)	Value	C3 vs C4 (diabetes prevalence)	Value
df	10	df	47	df	14	df	10	df	15	df	11
P(T < =t) one-tail	4.45X10^−01^	P(T < =t) one-tail	6.14X10^−09^	P(T < =t) one-tail	1.06X10^−02^	P(T < =t) one-tail	6.74X10^−02^	P(T < =t) one-tail	3.24X10^−09^	P(T < =t) one-tail	2.39X10^−07^
P(T < =t) two-tail	8.89X10^−01^	P(T < =t) two-tail	1.23X10^−08^	P(T < =t) two-tail	2.12X10^−02^	P(T < =t) two-tail	1.35X10^−01^	P(T < =t) two-tail	6.49X10^−09^	P(T < =t) two-tail	4.78X10^−07^

*Note:* Welch’s test was performed to determine statistical significance.

### SVM classifier model construction and optimisation

**Figure 4.**
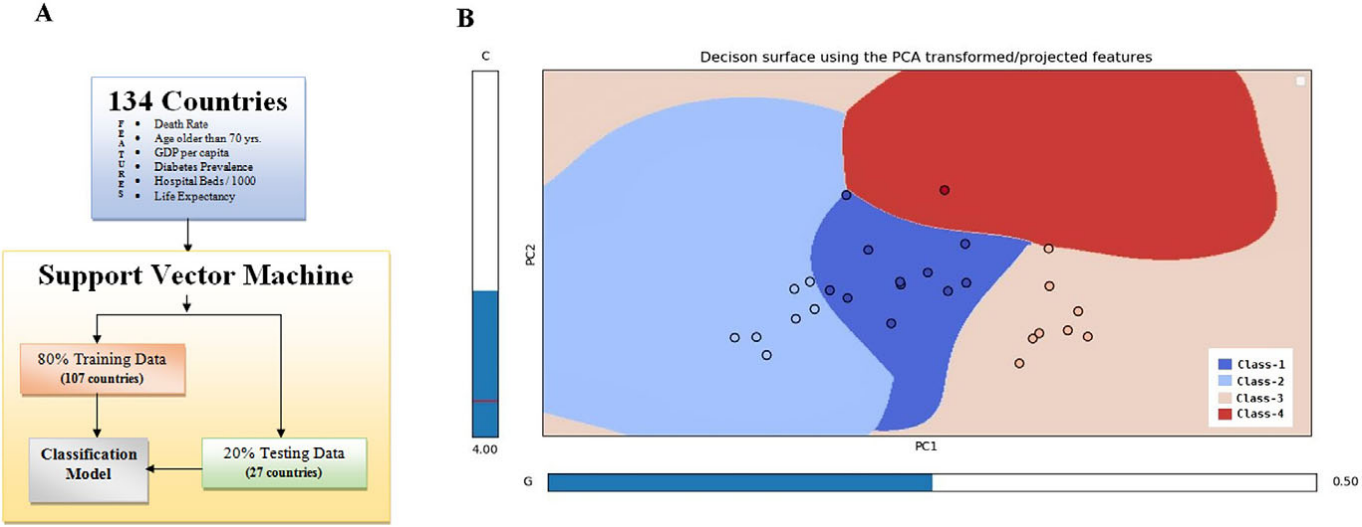
Supervised machine-learning approach to classify selected countries. Workflow for support vector machine (SVM) (a). Decision surface showing different class boundaries using the model and test data set (b).

**Figure 5.**
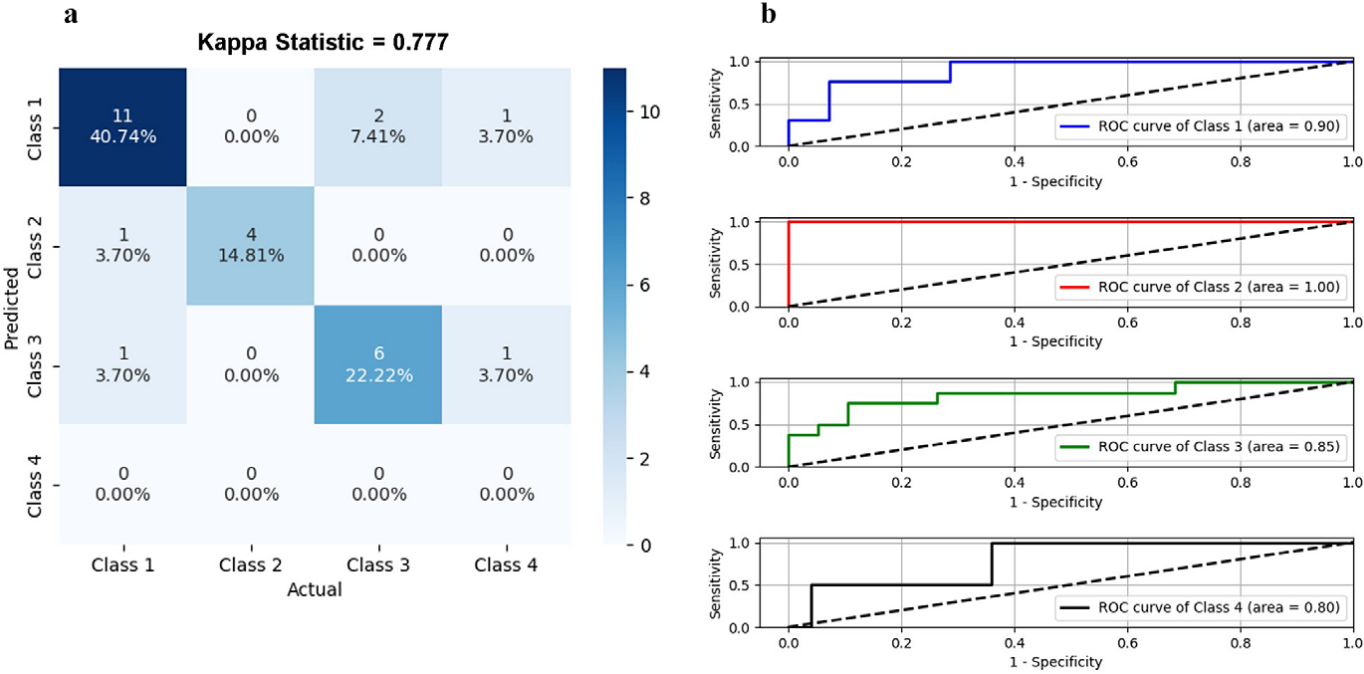
Performance of SVM-based classifier model. Confusion matrix showed that most countries in the test data set were classified precisely (a). Receiver operating characteristic (ROC) curves for the SVM classifier exemplify the predictive powers of classes 1, 2, 3, and 4, respectively. Areas under the curves (AUCs) of the four ROC curves are indicated accordingly (b).

Our study uses a data set with six features from 134 countries and follows the Pareto principle using an 80/20 split for training and testing. This rule, based on wealth distribution, is useful for explaining human and machine phenomena. The train–test split data (107 countries and 27 countries) are useful in estimating the performance of our SVM model when asked to make predictions on data not used to train the model (Figure 4a). We have used the Gaussian kernel radial basis function (RBF) to add a radial basis to improve the transformation. To trade off the misclassification of training examples against the simplicity of the decision surface, we have kept ‘C’ as 1. A low C makes the decision surface smooth, while a high C aims at classifying all training examples correctly. We keep the gamma hyper-parameter as 1, to keep optimum curvature at the decision boundary. Our SVM model is plotted across principal components 1 and 2 generated from our data. In the plot, four distinct colours represent four clusters based on the training data set. The dots represent the testing data set predictions. The colours of the dots represent the actual cluster they originally belonged to. The location of the dots shows the cluster where the SVM model has predicted the data to be part of (Figure 4b). Kappa statistics beautifully deals with data that are the result of a judgement, not a measurement. In our study, the kappa statistics value of 0.777 lies within the range of [0, 1], showing a substantial or strong level of agreement between the actual and predicted cluster values for all the test data (Figure 5a). Eleven countries were correctly identified for cluster 1, six countries for cluster 3, and four countries for cluster 2. Two data each from clusters 1, 3, and 4 were wrongly identified. The receiver operating characteristic (ROC) curve plotted for all four clusters shows an area > 80%, showing the promising outcome and usefulness of this SVM model in general (Figure 5b). ROC for cluster 2 comes out as a perfect classifier with an area equal to 1.00, followed by cluster 1 with an area of 0.90. Next is cluster 3, with an area equal to 0.85. Cluster 4 has the least area of 0.80.

## Discussion

Any disease like COVID-19, which has a global impact with a deadly outcome, always attributed to a comprehensive range of factors. The various factors influencing the spread and severity of the disease can be gathered through various sources such as health records, surveys, and data analysis. Initially, we used K-means clustering analysis and principal component analysis (PCA) to group countries into four distinct clusters based on a six-parameter scale. By grouping countries into clusters, based on similar characteristics, it becomes easier to identify patterns and relationships between the variables.

In addition, we employed an SVM classifier model to make predictions about which cluster each country would belong to. SVM is a supervised learning algorithm commonly used for classification tasks. Using the SVM model based on the six parameters, we were able to accurately predict which cluster each country belonged to, showing a high level of agreement between the actual and predicted cluster values. To evaluate the performance of our classifier model, we used the ROC curve, which plots the true-positive rate against the false-positive rate. The high ROC area values of 1.00, 0.90, 0.85, and 0.80 for the different clusters suggest that our classifier had a high degree of accuracy in assigning countries to their respective clusters. Overall, our study demonstrates the utility of clustering and SVM classification in identifying factors that influence the spread and severity of diseases such as COVID-19. Life expectancy and death rate are two significant measures that describe the overall health of a population. Our research indicates that these two parameters are inversely related – higher life expectancy shows a lower death rate, while lower life expectancy demonstrates a higher death rate. Our study also showed that life expectancy and hospital beds per thousand people have a positive correlation, although it is not a very strong one. Life expectancy is influenced by various factors such as genetics and economic status [34]. Adequate access to health care can have a positive impact on life expectancy. However, the availability of a high number of hospital beds per thousand people can ensure that individuals have access to essential medical care, reducing the risk of death [35]. However, other factors such as the quality of medical care and the general health of the population can also affect death rates, so hospital beds per thousand should not be considered the sole indicator of a country’s death rate. Older adults, particularly those over the age of 70, are more prone to health complications associated with COVID-19 and may require hospitalisation [16]. A high number of hospital beds per thousand can provide better support and care to older adults in a country. The death rate and age of 70 in a country are related as older individuals have a higher mortality rate due to health complications. However, our research showed an exception to this due to factors such as a strong GDP per capita, a sufficient number of hospital beds per thousand, and high life expectancy. Diabetes is a chronic condition that can increase the risk of health complications and death, especially if it is not properly managed. Studies show that individuals with diabetes are more susceptible to severe illness from COVID-19 and other infectious diseases [18]. Although diabetes prevalence and death rate are not directly related, they can provide a broader understanding of a population’s health when used together. Diabetes prevalence, life expectancy, and age of 70 in a country can be related as higher diabetes prevalence is more common in older adults, particularly those over the age of 70. Higher rates of diabetes can lead to health complications and decreased life expectancy, while areas with higher life expectancy may have lower rates of diabetes. However, the relationship between diabetes prevalence and life expectancy can be impacted by other factors such as access to healthcare and healthy lifestyle practices. The relationship between diabetes prevalence and GDP per capita is complex and depends on several factors, including lifestyle and government policies. Generally, a higher GDP per capita indicates better access to medical services, which may lower diabetes prevalence [36]. However, lifestyle factors such as unhealthy diets and lack of physical activity, which are often associated with higher GDP, can contribute to higher diabetes prevalence in some countries. Further studies and analysis are required to better understand the relationship between diabetes prevalence and GDP per capita. GDP per capita and hospital beds per thousand can serve as indicators of a country’s health system and its ability to provide medical services. Typically, higher GDP per capita is associated with better medical infrastructure and more resources to provide better health services, including more hospital beds. This can actually reduce the death burden associated with pandemics like COVID-19.

## Conclusion

There are several factors that have been found to influence the spread and severity of COVID-19.

We used K-means cluster analysis and principal component analysis to group countries into four distinct clusters based on six parameters and further developed the SVM classifier model to make predictions. Results showed a high degree of accuracy in assigning countries to their respective clusters. Life expectancy and death rate were found to be inversely related and positively correlated with hospital beds per thousand people. Diabetes prevalence was related to life expectancy and older age, and its relationship with GDP per capita was complex and dependent on several factors. GDP per capita and hospital beds per thousand were indicators of a country’s health system and ability to provide medical services. In conclusion, the study sheds light on the various factors that affect the spread and severity of COVID-19, highlighting the importance of access to healthcare and medical services in reducing death rates.

## Data Availability

The data related to the study are available from the corresponding author upon reasonable request.
